# Helical Rim Reconstruction: Antia-Buch Flap

**Published:** 2015-10-08

**Authors:** Chung Stella, Feintisch Adam M., Lee Edward

**Affiliations:** Division of Plastic and Reconstructive Surgery, Department of Surgery, New Jersey Medical School, Rutgers University, Newark

**Keywords:** Antia-Buch, helical rim, auricular reconstruction, helical rim defects, chondrocutaneous advancement flap

**Figure:**
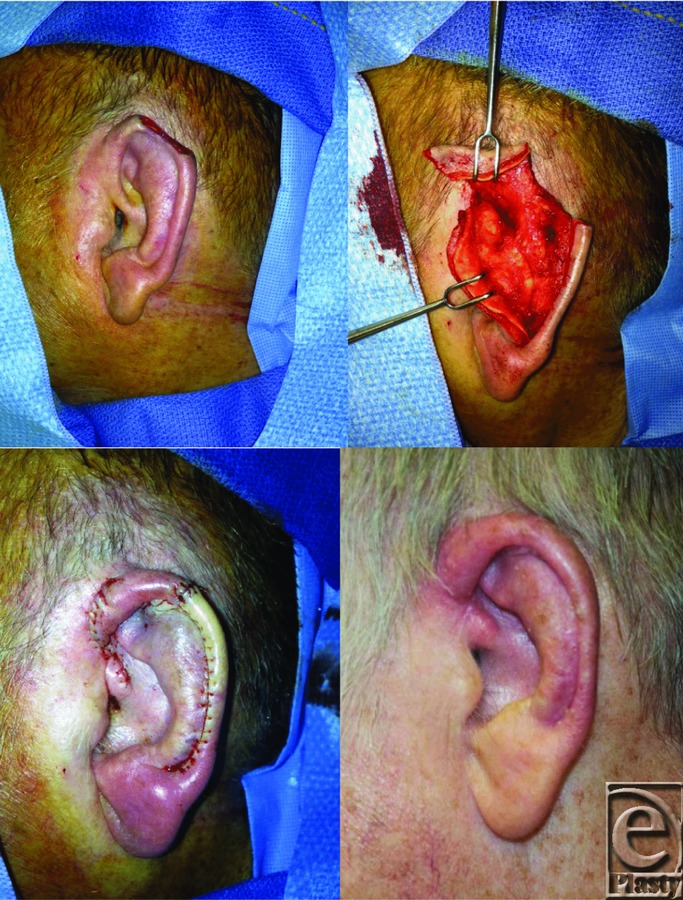


## DESCRIPTION

A 67-year-old male patient presented with basal cell carcinoma of the superior helix of the left ear. He underwent Mohs surgery with complete tumor excision resulting in an approximately 2.5 cm open wound with exposed cartilage. The defect was reconstructed with an Antia-Buch chondrocutaneous advancement flap.

## QUESTIONS

1. **How are auricular defects, in particular helical rim defects, classified?**
2. **What are the anatomical and aesthetic relationships of the ear?**
3. **What are the reconstructive options for helical rim defects?**
4. **What is an Antia-Buch chondrocutaneous advancement flap?**

## DISCUSSION

Auricular defects are classified as either partial- or full-thickness. A particular defect is further described by its anatomical location: helix, superior, middle, or inferior third of the auricle, and lobule. In the helical rim, full-thickness defects less than 1.5 cm are categorized as small, 1.5 to 2 cm as medium, and greater than 2 cm as large. When reconstructing a helical rim defect, one must have an accurate understanding of the deformity and approach the repair systematically.^1^ Vascular supply to the ear, tumor recurrence, cosmesis, and restoration of function (ie, support for hearing aids and eyeglasses) must be thoroughly discussed between the surgeon and the patient.

The ear is trisectioned by its cartilaginous framework of conchal, antihelix-antitragus, and helix-lobule complexes. The auricular blood supply is from branches off the external carotid, namely, the posterior auricular and superficial temporal arteries. The sensory supply to the ear is derived from contributions of the auriculotemporal, great auricular, lesser occipital, vagus, and glossopharyngeal nerves. Knowledge of this anatomy facilitates proper blockage using local anesthetics. The ear approaches 85% of its adult size by 3 years of age, with complete maturation by early adolescence. As one ages, cartilage calcification increases with resultant stiffness. Certain anatomical landmarks must also be appreciated to achieve an acceptable reconstruction. The auricle is located one ear-height distance from the lateral orbital rim and aligns with the nasal ala and lateral brow, slanting 21° to 25° outward. The helix to mastoid distance is normally 10 to 12 mm in the upper third, 16 to 18 mm in the middle third, and 20 to 22 mm in the lower third. In addition to preserving a smooth, gently curved, and well-defined antihelical fold, the long axis of the ear should be posteriorly inclined approximately 20° with maintenance of a conchoscaphal angle of around 90°.^2^

There are various methods of surgical repair of helical rim defects. Important factors to consider when choosing a reconstructive method are size and complexity of the wound, exposed structures, and whether local tissues are available. Small defects can usually be closed primarily with various wedge resections. For medium-sized defects, chondrocutaneous advancement flaps, rotation flaps, or contralateral composite grafts can be used. Large defects typically need bipedicled tubed flaps or a temporoparietal fascial flap with skin and cartilage grafts.

The Antia-Buch chondrocutaneous advancement flap is ideal for small- to medium-sized helical defects. This surgical technique may also be used with larger defects when a helical crus V-Y advancement is incorporated into the design.^3^ The Antia-Buch flap is designed by making an incision along the helical sulcus extending through the anterior skin and cartilage, dissecting the helix and scapha free from one another. The posterior auricular skin is elevated superficial to the perichondrium. The resulting anterior chondrocutaneous flap is then advanced into the defect. If extra length is needed, a V-Y advancement of the helical crus and trimming of the scaphal cartilage may reduce tension at the reapproximated wound edges.^4^ The Antia-Buch flap reconstruction can be accomplished in a single stage. For deformities greater than 2.8 cm, auricular distortions may result with this flap and a variety of other reconstructive options can be used.

The Antia-Buch flap reconstruction is a simple and convenient technique that gives a superior aesthetic auricular appearance. Anatomical landmarks are restored, whereas scars are concealed in the natural concavities and convexities of the ear.^5^ Although reduction in total ear circumference and vertical height may be appreciated, it is typically minimal in nature.^6^ In such cases, additional reconstructive modalities or a modified Antia-Buch repair may be considered.^7^

Our patient successfully underwent a standard Antia-Buch flap reconstruction with a helical crus V-Y advancement for closure of a 2.5 cm open wound of the superior helix. One month postoperatively, the patient's incisions were well healed and his natural auricular aesthetics were restored.

## References

[1] Brent B (1977). The acquired auricular deformity: a systematic approach to its analysis and reconstruction. Plast Reconstr Surg.

[2] Salgarello M, Visconti G (2013). Combined Technique in Aesthetic Otoplasty in Advanced Cosmetic Otoplasty.

[3] Crikelair GF (1956). A method of partial ear reconstruction for avulsion of the upper portion of the ear. Plast Reconstr Surg.

[4] Thorne CH, Beasley RW, Aston SJ, Bartlett SP, Gurtner GC, Spear SL (2007). Specific regional defects. Grabb and Smith's Plastic Surgery.

[5] Low DW (1998). Modified chondrocutaneous advancement flap for ear reconstruction. Plast Reconstr Surg.

[6] McRae MC, Au A, Narayan D (2009). A novel method of auricular reconstruction. Ann Plast Surg.

[7] De Schipper H, van Rappard J, Dumont E (2012). Modified Antia Buch repair for full-thickness middle auricular defect. Dermatol Surg.

